# Leveraging Comprehensive Echo Data to Power Artificial Intelligence Models for Handheld Cardiac Ultrasound

**DOI:** 10.1016/j.mcpdig.2025.100194

**Published:** 2025-01-10

**Authors:** D.M. Anisuzzaman, Jeffrey G. Malins, John I. Jackson, Eunjung Lee, Jwan A. Naser, Behrouz Rostami, Grace Greason, Jared G. Bird, Paul A. Friedman, Jae K. Oh, Patricia A. Pellikka, Jeremy J. Thaden, Francisco Lopez-Jimenez, Zachi I. Attia, Sorin V. Pislaru, Garvan C. Kane

**Affiliations:** Department of Cardiovascular Medicine, Mayo Clinic, Rochester, MN

## Abstract

**Objective:**

To develop a fully end-to-end deep learning framework capable of estimating left ventricular ejection fraction (LVEF), estimating patient age, and classifying patient sex from echocardiographic videos, including videos collected using handheld cardiac ultrasound (HCU).

**Patients and Methods:**

Deep learning models were trained using retrospective transthoracic echocardiography (TTE) data collected in Mayo Clinic Rochester and surrounding Mayo Clinic Health System sites (training: 6432 studies and internal validation: 1369 studies). Models were then evaluated using retrospective TTE data from the 3 Mayo Clinic sites (Rochester, n=1970; Arizona, n=1367; Florida, n=1562) before being applied to a prospective dataset of handheld ultrasound and TTE videos collected from 625 patients. Study data were collected between January 1, 2018 and February 29, 2024.

**Results:**

Models showed strong performance on the retrospective TTE datasets (LVEF regression: root mean squared error (RMSE)=6.83%, 6.53%, and 6.95% for Rochester, Arizona, and Florida cohorts, respectively; classification of LVEF ≤40% versus LVEF > 40%: area under curve (AUC)=0.962, 0.967, and 0.980 for Rochester, Arizona, and Florida, respectively; age: RMSE=9.44% for Rochester; sex: AUC=0.882 for Rochester), and performed comparably for prospective HCU versus TTE data (LVEF regression: RMSE=6.37% for HCU vs 5.57% for TTE; LVEF classification: AUC=0.974 vs 0.981; age: RMSE=10.35% vs 9.32%; sex: AUC=0.896 vs 0.933).

**Conclusion:**

Robust TTE datasets can be used to effectively power HCU deep learning models, which in turn demonstrates focused diagnostic images can be obtained with handheld devices.

Echocardiography is the most widely employed imaging modality for evaluating cardiac structure and function.^1^ However, comprehensive transthoracic echocardiography (TTE) requires extensive training to gain the skills needed for correct performance and interpretation; image quality can vary depending on the operator, ultrasound machine, and patient characteristics; and there can be interobserver variability in study interpretation.^1^ Handheld cardiac ultrasound (HCU), in which handheld ultrasound devices facilitate focused cardiovascular ultrasound imaging in point-of-care settings such as the bedside,^2^ can be performed by clinical providers without extensive training. However, because operators typically have limited experience, study interpretation for HCU can be even more variable than it is for TTE. In addition, HCU image quality can be more variable due to limitations in the technical capabilities of handheld devices, such as fewer transducer crystals, lower frame rates, absence of harmonic imaging, and lack of electrocardiography-gating.

Artificial intelligence (AI) approaches, using deep learning algorithms, may be well-suited to reduce variability for TTE and HCU.^1^^,^^3^, ^4^, ^5^ However, training deep learning algorithms requires large quantities of data, and this requirement poses an issue for HCU, as extant databases are not currently available for this nascent technology. As such, our goal was to develop a deep learning framework that leverages thousands of pre-existing diagnosed TTE studies to power both TTE and HCU AI.

When developing this framework, as a proof-of-concept, we chose to focus on 1 task that humans can do and 2 tasks that humans cannot do. Namely, for the task that humans can do, we chose to develop a model to estimate left ventricular ejection fraction (LVEF)^6^, as this is a key echocardiographic measurement that can benefit from potential reduction in variability. In particular, we aimed to develop a deep learning framework that can estimate LVEF from both TTE and HCU videos in a fully end-to-end fashion—without manual labeling of cardiac views and without segmentation of cardiac structures—taking as input unlabeled Digital Imaging and Communications in Medicine (DICOM) clips and outputting an LVEF estimate. For the tasks that humans cannot do, we elected to develop AI models to estimate patient age and sex.^7^, ^8^, ^9^

By developing an end-to-end deep learning framework, which can rapidly be expanded to estimate other clinical measurements, patient characteristics, and cardiac pathologies, we aimed to build a set of tools that could potentially optimize deployment of cardiac imaging resources by reducing the need for formal diagnostic echocardiography and assist point-of-care providers with limited experience in interpreting echocardiographic examinations.

## Patients and Methods

### Ethics Statement

All study procedures were approved by the Mayo Clinic institutional review board, and patient cohorts only included individuals who had provided previous authorization for the use of their data in research. Study data were collected between January 1, 2018 and February 29, 2024.

### Data Acquisition and Selection

#### Retrospective Cohorts

For model training, we first retrieved the list of all adult patients at Mayo Clinic Rochester or within the Mayo Clinic Health System (MCHS) whose most recent rest TTE study was between January 1, 2018 and December 31, 2021, taking the most recent examination if a patient had more than one within this date range. We excluded patients whose data were part of the training set for the view classifier embedded in our data processing workflow.^10^ We then randomly sampled from the remaining list (additional details provided in the Supplemental Appendix, available online at https://www.mcpdigitalhealth.org/).

Clinically indicated echocardiography was performed on GE Healthcare E95 or Philips Epiq machines and recorded in clips of 3 cardiac cycles. The reference measured LVEF was on the basis of the clinical measure using the following hierarchy^11^: 3D volumes, 2D biplane, 2D modified Quinones, M-mode modified Quinones, and lastly, a visual estimate by a level III trained expert echocardiographer.

For each TTE study, DICOM data were downloaded and preprocessed as described in the Supplemental Appendix. Next, we identified studies with at least 1 video passing view selection criteria for each of the following 3 views: parasternal long axis (PLAX), apical 4-chamber (A4C), and apical 2-chamber (A2C). These studies were divided into datasets for training (70%), validation (15%), and testing (15%) using the train test split function in the scikit-learn library ^12^ in Python version 3.9.6.^13^ Note that the validation dataset was used to make decisions regarding model architecture as well as hyperparameter tuning. In addition to the testing dataset from Rochester/MCHS, we also evaluated model performance for the LVEF model using randomly sampled sets of TTE studies performed at the Arizona and Florida sites of Mayo Clinic between January 1, 2022 and February 29, 2024.

#### Prospective Cohort

HCU data were collected from a prospective cohort of patients who visited Mayo Clinic Rochester as an outpatient for a clinically indicated TTE examination from November 1, 2022, to September 30, 2023. Immediately after their TTE examination (ie, within the same session in the same environment), if patients provided verbal consent for additional clips to be collected for research purposes, the same sonographer who performed the TTE examination collected the following 5 2D video clips using a HCU device (Lumify, Philips Healthcare): PLAX, parasternal short axis, A4C, apical 3-chamber, and A2C. After downloading and preprocessing data (as described in the Supplemental Appendix), we identified patients passing view selection criteria for both TTE and HCU.

### Deep Learning Framework and Model Architecture

**Figure 1:**
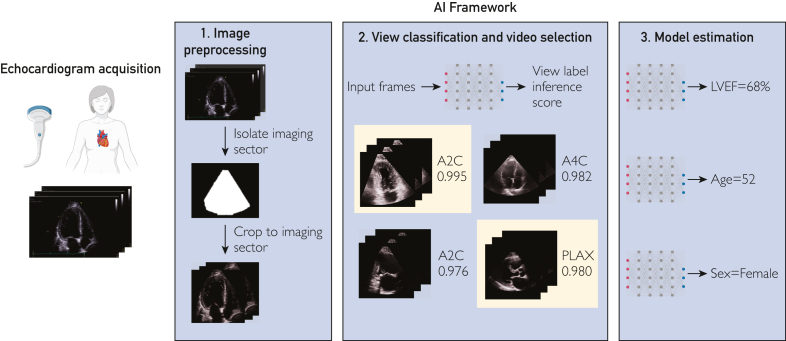
The end-to-end deep learning framework employed in the current study. By including image preprocessing, view classification, and deep learning models for LVEF, age, and sex estimation, the current framework can be considered fully end-to-end. A hypothetical example is shown in the figure; as such, the view classifier inference scores and model estimates do not correspond to actual data points. The videos highlighted in yellow denote those that would be selected in this example (ie, those with the highest inference score for the A2C and PLAX views). The icons used to denote the ultrasound probe, patient, and deep learning networks were taken from BioRender.com. A2C, apical 2-chamber; A4C, apical 4-chamber; PLAX, parasternal long axis.

As shown in Figure 1, the deep learning framework included preprocessing steps such as identification of the echocardiographic imaging sector, followed by using a deep learning model to classify the views from which echocardiographic videos were obtained,^10^ and finally LVEF (or age or sex) estimation using a deep learning model (one for each task). The deep learning models for estimating LVEF, age, and sex had 3D convolutional neural networks as the main backbone for feature extraction from echocardiographic videos. Additional details regarding model architecture are provided in the Supplemental Appendix.

### Model Training and Hyperparameters

We selected 48 frames to constitute a single input unit (video). The selection of this value, which was considered a hyperparameter, was guided by internal validation using several values (eg, 32, 40, and 48), and took performance and hardware limitations into account. This window of 48 frames typically represents 1.6 seconds in a 30 frames-per-second video. On the basis of the number of available frames, we selected up to 3 sliding windows from each training video (frames 1-48, 25-72, and 49-96). For multi-view models, because videos were passed as a collection of inputs, both (or all 3) videos from a patient’s examination had to meet a minimum frame requirement to be included in the training dataset. Additional details regarding model training are provided in the Supplemental Appendix.

### Data Augmentation

To increase model generalizability, we applied 4 augmentations to the training dataset: random rotation between −10 and +10 degrees, Gaussian blurring, central cropping, and random cropping.^14^ Random rotation was applied because we observed that due to variation in probe placement during data acquisition, the echocardiographic imaging sector (and cardiac anatomy) was sometimes not centered at the middle of each video frame; Gaussian blurring was applied to make the model more robust for lower quality echocardiographic videos; central and random cropping were applied because we observed that on some videos, some portions of the heart chambers were cropped (additional details provided in the Supplemental Appendix).

### Model Selection

To estimate LVEF, we initially trained the following models: 3 single view models using A2C, A4C, and PLAX views as input; three 2-view models trained with the respective combinations of A2C and A4C, A2C and PLAX, and A4C and PLAX views; and a 3-view model trained with A2C, A4C, and PLAX views. Though we hypothesized the 3-view model would perform best, when we evaluated all 7 models for LVEF estimation using the validation dataset, we observed that, likely due to poor performance of the model on A4C videos, the A2C and PLAX model instead showed the best performance. Therefore, we selected the A2C and PLAX model for all further testing in terms of LVEF and also used the same views for the age estimation and sex classification models (additional details provided in the Appendix).

### Evaluating Model Performance

For LVEF estimation, we first performed continuous regression using tumbling sliding windows. For each examination, we estimated LVEF from a maximum of 6 windows (frames 1-48, 13-60, 25-72, 37-84, 49-96, and 61-108)—depending on the available number of frames from A2C and PLAX videos—and then computed an overall average estimate across windows. Second, we performed a binary classification between significantly reduced LVEF (clinically calculated LVEF ≤40%) versus normal or mildly reduced LVEF (clinically calculated LVEF >40%). We also performed a binary classification between clinically calculated LVEF ≤50% versus clinically calculated LVEF >50%. To perform these analyses, we obtained optimal cut-point LVEF values from the receiver operating characteristic (ROC) curve for the validation dataset (for the 40% threshold and for the 50% threshold) by selecting the values that minimized the Euclidean distances between each respective ROC curve and the (0,1) point.^15^ We then applied these thresholds (49.31% for 40% threshold and 55.26% for 50% threshold) to the testing datasets, which allowed us to account for potential systematic biases such as a tendency to overestimate LVEF. For age, we performed a continuous regression with tumbling windows exactly as described for LVEF, and for sex, we performed a binary classification between male and female.

## Results

### Sample Characteristics

Models were developed using TTE data from 7801 patients (1 study per patient; 6432 patients for training and 1369 for internal validation). Models were then evaluated using retrospective TTE data from the 3 Mayo Clinic sites (Rochester, n=1970; Arizona, n=1367; and Florida, n=1562) and a prospective cohort of 625 patients from whom TTE and HCU data were simultaneously collected. As shown in Table 1, although the mean LVEF was lower in the model development cohorts from Rochester/MCHS (by design) than the Arizona and Florida cohorts and the prospective cohort, the distributions of patient age and sex were fairly comparable across all cohorts.

**Table 1:** Demographic and Clinical Characteristics of Patient Cohorts

	Retrospective TTE Cohorts From Rochester/MCHS			Retrospective TTE Cohorts From Other Sites		Prospective TTE and HCU Cohort	
	Training	Validation	Testing	Arizona	Florida		
Number of studies	6432	1369	1970	1367	1562	625	
Age on study date (y)							
Mean ± SD	65±16	65±17	66±17	69±15	66±15	64±16	
Range	18-106	18-100	18-104	19-103	18-100	18-96	
Sex (%)							
Female	42.02	42.00	43.23	40.01	44.62	39.52	
Male	57.98	58.00	56.77	59.99	55.38	60.48	
Race and ethnicity (%)							
NH American Indian/Alaska Native	0.37	0.29	0.36	1.61	0.13	0.48	
NH Asian	1.51	1.31	0.81	3.66	2.24	2.08	
NH Black/African American	2.04	1.90	2.23	2.85	10.31	2.40	
Hispanic/Latino	2.38	1.83	2.54	8.92	5.89	1.44	
NH Native Hawaiian/Other Pacific Islander	0.03	0.15	0.15	0.22	0.13	–	
NH White	89.47	90.28	90.10	80.25	78.68	91.20	
Other or unknown	4.20	4.24	3.80	2.49	2.62	2.40	
Left ventricular ejection fraction							
Mean ± SD	54±12	54±12	54±13	59±10	60±10	57±11	
Range	8-82	9-78	10-79	12-79	6-82	11-75	
Comorbidities (%)							
Systemic hypertension	59.20	59.31	65.33	64.67	63.83	56.96	
Congestive heart failure	36.72	36.01	40.86	34.02	33.16	42.08	
Valvular disease	38.46	41.27	41.17	59.77	49.94	54.56	
Peripheral vascular disease	38.48	36.82	41.57	41.55	38.48	46.40	
Chronic pulmonary disease	31.23	31.85	32.74	24.65	25.80	24.80	
Diabetes mellitus	23.79	21.55	27.36	21.43	26.06	18.88	
Renal disease	25.70	24.11	30.71	29.55	29.45	25.92	
Liver disease	16.29	14.17	16.55	17.22	21.19	16.64	
Orientation of A4C videos (%) Left side of heart on left of image Right side of heart on left of image	83.68 16.32	85.83 14.17	74.06 25.94	100 –	100 –	TTE	HCU
						100 –	100 –
Equipment manufacturer (%) GE Healthcare Philips Medical Systems EchoNous	89.78 10.20 0.02	90.36 9.64 –	85.53 14.47 –	99.63 0.37 –	97.44 2.56 –	TTE	HCU
						100 –	– 100

Binary sex and race and ethnicity were self-reported and obtained from each patient’s medical record. Comorbidities were ascertained using the comorbidity package in R^37^ on the basis of ICD-9 or ICD-10 codes noted in the patient’s medical record either on or before the echocardiographic study date.

Abbreviations: A4C, apical 4-chamber; MCHS, Mayo Clinic Health System; NH, non-Hispanic.

### LVEF Estimation Model

#### LVEF Estimation for the Retrospective Cohorts

**Figure 2:**
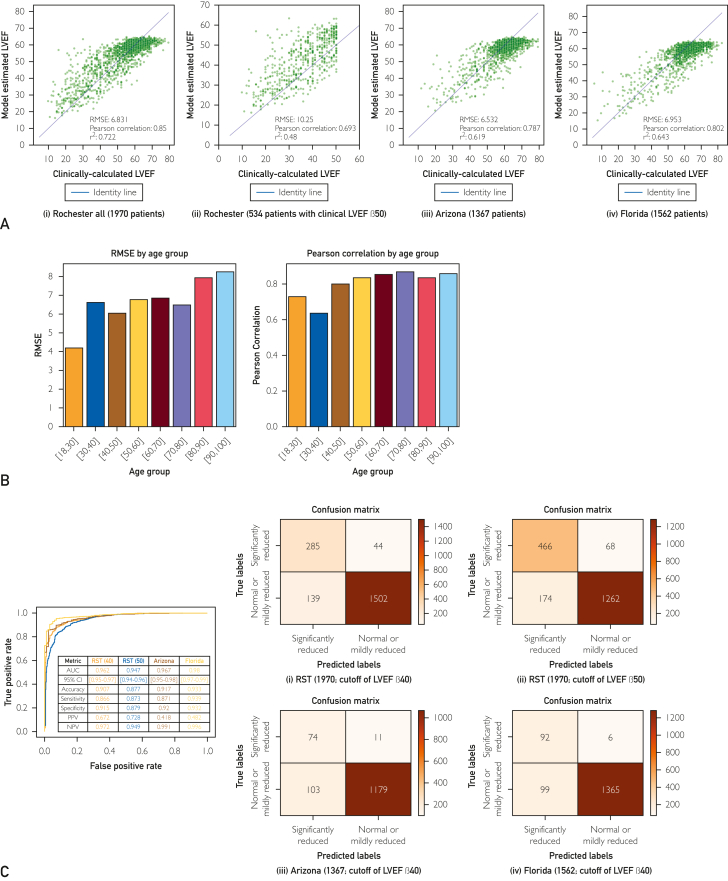
LVEF model performance for the retrospective testing dataset of TTE data. (A) Scatterplot correlations of clinically calculated versus model-estimated LVEF for (i) all patients in the Rochester cohort, (ii) patients in the Rochester cohort with clinically calculated LVEF ≤ 50, (iii) all patients in the Arizona cohort, and (iv) all patients in the Florida cohort. (B) Model performance comparison for specific age subgroups. (C) LVEF binary classification performance. Significantly reduced denotes LVEF ≤ 40% (for the confusion matrices in [i], [iii], and [iv]) and LVEF ≤ 50% (for the confusion matrix in [ii]), whereas normal or mildly reduced denotes LVEF > 40% (for the confusion matrices in [i], [iii], and [iv]) and LVEF > 50% (for the confusion matrix in [ii]). AUC, area under the curve; NPV, negative predictive value; PPV, positive predictive value; RMSE, root mean squared error; RST, Rochester.

As shown in Figure 2A (i), the LVEF estimation model showed a correlation (*r*=0.850) between ground truth labels (clinically calculated LVEF from TTE) and model estimates for the Rochester cohort (all 1970 patients), which was reflected in a low RMSE of 6.831. In 87.26% cases, model estimates showed a difference of 10% or less from clinically calculated LVEF. This rate is comparable to that shown by expert clinicians.^16^ We also examined the correlation between ground truth labels and model estimates for the 534 patients with a clinically calculated LVEF ≤50 (Figure 2A [ii]). Although the RMSE was higher and the correlation strength weaker for this specific group (RMSE=10.25; *r*=0.693), model performance was still considerable.

As shown in Figure 2A (iii) and Figure 2A (iv), performance was also strong for both the Arizona (RMSE=6.532; *r*=0.787) and Florida (RMSE=6.953; *r*=0.802) datasets. This was observed even though the model was never exposed to data from either of these sites during training.

In Figure 2B, the LVEF model performance is detailed for specific age subgroups. Although there was some variability in performance across subgroups (likely due to representation in the sample, which was poorer for individuals in the extreme younger and older age subgroups), performance was nevertheless quite strong across the board (eg, Pearson correlation coefficients above 0.6 for all subgroups), suggesting that LVEF performance is not contingent on detecting patient age.

Finally, results for binary classification of LVEF are shown in Figure 2C. The area under the curve (AUC) using a cutoff of clinically calculated LVEF ≤40% was 0.962 (95% CI: 0.95-0.97) for the Rochester cohort, 0.967 (95% CI: 0.95-0.98) for the Arizona cohort, and 0.980 (95% CI: 0.97-0.99) for the Florida cohort. When we instead used a cutoff of clinically calculated LVEF ≤50%, the AUC for the Rochester cohort was 0.947 (95% CI: 0.94-0.96).

#### LVEF Estimation for the Prospective Cohort

**Figure 3:**
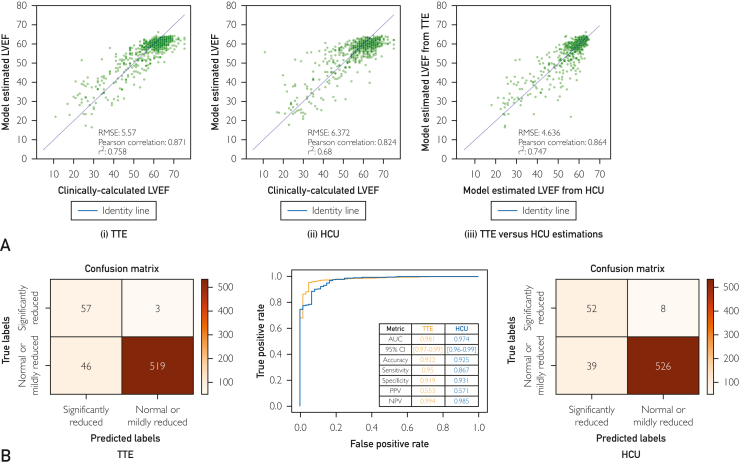
(A) Comparison of LVEF estimation model performance for transthoracic echocardiography (TTE) (i) versus handheld cardiac ultrasound (HCU) data (ii) from the prospective cohort, as well as the correlation between model estimates for TTE versus HCU (iii). (B) LVEF binary classification performance for HCU and TTE data from the prospective cohort. AUC, area under the curve; NPV, negative predictive value; PPV, positive predictive value; RMSE, root mean squared error.

As shown in Figure 3A, model performance was comparable between TTE (RMSE=5.570; *r*=0.871) and HCU examinations (RMSE=6.372; *r*=0.824). The difference in RMSE between TTE and HCU was minimal (0.802%), and a strong correlation (*r*=0.864) was observed when model estimates for TTE versus HCU examinations were directly compared (Figure 3A [iii]). As shown in Figure 3B, for the LVEF binary classification (LVEF cutoff of ≤40%), we observed comparable results between TTE and HCU examinations for all quantified metrics.

### Age Estimation and Sex Classification Models

**Figure 4:**
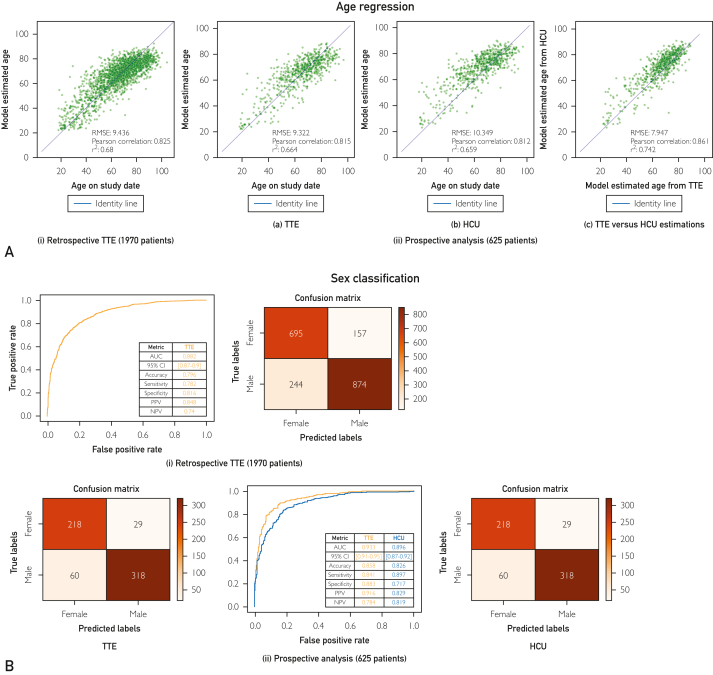
(A) Age estimation results. (i) Correlation between chronological age and model-estimated age for the testing dataset of retrospective TTE data from Mayo Clinic Rochester. (ii) For the prospective cohort, correlation between chronological age and model-estimated age for TTE (a) and HCU (b), and the correlation between model-estimated age for TTE versus HCU (c). (B) Sex classification performance for the testing dataset of retrospective TTE data from Mayo Clinic Rochester (i), and comparison between TTE and HCU data from the prospective cohort (ii). For this analysis, male is considered the positive class. AUC, area under the curve; NPV, negative predictive value; PPV, positive predictive value

Figure 4A details model performance for age estimation. As shown in the figure, we observed a strong correlation between chronological age and model-estimated age for the testing dataset of the retrospective cohort (RMSE=9.436; *r*=0.825). For the prospective cohort, model performance was comparable between TTE (RMSE=9.322; *r*=0.815) and HCU examinations (RMSE=10.349; *r*=0.812). The difference in RMSE between TTE and HCU was minimal (1.03 years), and a strong correlation (*r*=0.861) was observed when model estimates for TTE versus HCU examinations were directly compared (Figure 4A [ii]-[c]).

Figure 4B details model performance for sex classification. Here, we also observed strong performance for the testing dataset of the retrospective cohort (AUC=0.882) and comparable results between TTE and HCU examinations in the prospective cohort across all quantified metrics.

## Discussion

HCU is poised to transform medicine by enabling point-of-care diagnosis of potentially critical conditions, but human image interpretation requires substantial training and expertise. We sought to leverage an extant large TTE dataset to demonstrate the ability of an AI deep learning framework to automatically analyze TTE and to utilize this rich TTE dataset to enable AI to interpret HCU images. As a proof-of-concept, we evaluated the performance of this framework on estimations of the following parameters: (1) LVEF, (2) patient age, and (3) patient sex. We first trained AI models using retrospective TTE data, and then prospectively applied these models to TTE and HCU data collected from the same cohort of patients. In this prospective cohort—which is the largest of its kind to date—we observed strong model performance across the LVEF, age, and sex estimation models, with model estimates comparable between HCU and TTE videos.

### Estimating LVEF

Although deep learning approaches have been previously developed to estimate LVEF using TTE data,^8^^,^^16^, ^17^, ^18^, ^19^, ^20^, ^21^, ^22^, ^23^, ^24^, ^25^ some have relied on segmentation of cardiac structures to estimate ventricular volumes before computing LVEF.^21^, ^22^, ^23^, ^24^, ^25^, ^26^ By training our model directly on LVEF, we averted the possibility of errors in LVEF computation due to segmentation errors.^27^ In addition, this study differed from some previous work^8^^,^^17^^,^^18^^,^^20^ in that our models made use of multiple cardiac views (ie, A2C and PLAX views) as opposed to a single view, and our framework was fully end-to-end^19^ by including the use of a deep learning model for echocardiographic view classification.^10^

In terms of performance for TTE data, we achieved a PC of 0.79-0.85, RMSE of 6.53-6.95, *r*^*2*^ of 0.62-0.72, and AUC of 0.96-9.98 across the Rochester, Arizona, and Florida datasets. Although it is difficult to perform a head-to-head comparison with existing studies because performance depends on many variables, such as dataset source and size, training method and modality, cardiac views, and so on, these values were similar to or slightly superior to those observed in previous studies. In the study by Ouyang et al,^18^ the study authors observed an *r*^*2*^ of 0.66-0.71 and an RMSE of 6.16-7.55 for a test dataset consisting of 1282 patients having only A4C clips. Tromp et al^26^ achieved a PC of 0.75-0.89 and an RMSE of 6.8-12.6 on their internal and external datasets for LVEF estimation. Lau et al^28^ developed a 3-D convolutional neural network model named DROID to automate standard measurements of left atrial and left ventricular structure and function, and for LVEF estimation, achieved an *R*^*2*^ of 0.74 and 0.69 on the C3PO and EchoNet-Dynamic datasets, respectively.

In terms of HCU data, similar to a few previous studies,^16^^,^^29^ we were able to successfully estimate LVEF without previous detection of endocardial boundaries. The current findings therefore differ from existing approaches, which require LV segmentation,^30^, ^31^, ^32^, ^33^ which can be especially error-prone for videos collected using handheld ultrasound devices.^31^ By directly estimating LVEF, the current model also differs from AI workflows for HCU data that require estimation of contraction coefficients in the longitudinal and radial directions before estimation of LVEF.^16^

In terms of performance for HCU data, we observed a PC of 0.82, RMSE of 6.37, *r*^*2*^ of 0.68, and AUC of 0.97 on our prospective dataset. These values are again in line with those observed in previous studies. In the study by Asch et al,^16^ the authors achieved an intraclass correlation of 0.86-0.94 using different views and their combinations for a test dataset consisting of 166 patients with uniformly distributed LVEF. EchoNet-POCUS^29^^,^^30^ observed an intraclass correlation of 0.85 and PC of 0.87 on a test set of 100 patients. Using another HCU integrated with LVivo software, Filipiak-Strzecka et al^31^ observed a PC of 0.92 between AI estimates and the calculated 3-D volume-based LVEF measurements in a test set of 112 patients.

Critically, the strong performance for LVEF estimation that we observed for HCU data was comparable to that observed using TTE videos collected from the same individuals in the same imaging session, using a model entirely trained on TTE data. That is, our LVEF estimation model performed well on HCU data even though it was never exposed to HCU data during training. The impact of this finding is relevant: it enables millions of previously acquired TTE studies to be used to train the interpretation of HCU studies in a frictionless manner, and although not tested here, the methodology may also be applicable for clips collected using handheld ultrasound devices in point-of-care settings (point-of-care ultrasound, or POCUS).

We acknowledge that the HCU data was collected by experienced sonographers in a controlled setting (ie, the same setting used for TTE data collection), and therefore may be of higher quality than is typical for point-of-care datasets. With that said, in addition to the high quality of the images in the handheld ultrasound dataset, we also attribute some of the success of LVEF estimation for HCU to specific aspects of our model training, such as explicit consideration of temporal variability in the cardiac cycle. Because of a lack of electrocardiography-gating, we were unable to ensure that each HCU clip began at the same point of the cardiac cycle (ie, as is done in TTE protocols, in which videos typically begin at the peak of an R-wave). To address this, we elected to use temporal sliding windows during model training, exposing the model to TTE clips beginning at many different points of the cardiac cycle. In addition, the use of multiple types of image augmentation, such as Gaussian blurring (ie, mimicking the lower resolution of HCU videos) may also have partially accounted for the model’s success with HCU videos. These aspects of the model training—as well as additional aspects of the design such as the use of multiple echocardiographic views as model input—may explain why this study was successful despite previous work showing only minimal success when applying an AI model entirely trained on TTE data to handheld ultrasound studies.^34^ With that said, we acknowledge that because we have not directly compared models trained with and without each of these specific aspects of the training protocol, we can only speculate on what contributed to the model’s success with HCU clips.

### Estimating Patient Age and Classifying Patient Sex

Beyond quantifying LVEF, our models also showed evidence of successful estimation of patient age and classification of patient sex. The observed results are similar in magnitude to previous work in terms of RMSE and AUC for both TTE^7^ and for HCU.^9^ These findings underscore the richness of biological data encoded in an ultrasound image, even when acquired with a handheld device. This in turn has implications for the development of future AI applications, such as models built to detect specific cardiac pathologies on the basis of HCU clips. Furthermore, similar to the LVEF estimation model, the models we developed were successful even though they were never exposed to HCU data during training, again highlighting the feasibility of the current approach of leveraging comprehensive TTE data to power AI models for HCU data.

### Study Limitations

The current results should be considered in light of several limitations. First, patient cohorts were not diverse in terms of race and ethnicity, and to mitigate potential bias,^7^ future work should focus on external validation across diverse patient cohorts. Second, as mentioned previously, the HCU dataset does not reflect the heterogeneity that is characteristic of point-of-care imaging data across settings and operators with varying levels of imaging experience. Of consequence, further testing is required to evaluate model performance for images collected by different operators spanning numerous point-of-care settings such as emergency departments and bedside for critically ill patients.^35^ Third, potential errors in view classification by the automatic framework might have impacted estimates of LVEF, age, or sex, as incorrect video clips could have been selected and subsequently presented as input to these downstream models. This may be one reason why we observed that for LVEF, the 2-view model for A2C and PLAX outperformed a 3-view model that also included A4C, as the A4C videos identified by the view classifier (ie, those with the highest inference score) may not have been optimal for LVEF estimation (eg, potentially focused on the atria or the right ventricle as opposed to the left ventricle). To address this limitation, a single model that can intake any view could be developed and tested. This approach would also avoid the limitation of requiring an A2C video for model presentation, as this view can be more difficult for novice users to acquire compared with other views such as the A4C view.^36^ Finally, videos were required to have at least 48 frames for patients’ examinations to be included in either cohort, which may have excluded individuals with a higher heart rate. Although the number of frames was treated as a hyperparameter and tuned using the validation dataset, future work could more systematically examine the impact of video duration on model performance or use methods such as looping shorter videos to meet temporal length requirements.

## Conclusion

A fully end-to-end deep learning framework trained on extant TTE data can robustly estimate LVEF, age, and sex from input videos collected using handheld cardiac ultrasound, with model performance that is comparable to that observed for TTE input videos collected from the same set of patients. These proof-of-concept findings report that the framework we developed, which can rapidly be expanded to estimate other clinical measurements, patient characteristics, and cardiac pathologies, has the potential to assist in point-of-care settings, which may be especially useful for clinicians without extensive training in echocardiographic data collection and those in resource-poor settings lacking sophisticated echocardiographic equipment.^2^ Even though this research is still in its initial stages, we are nevertheless encouraged by the potential promise of these results to inform patient care.

## Potential Competing Interests

Drs Anisuzzaman, Malins, Jackson, Lee, Naser, Pislaru, Kane, and Attia. have invented algorithms licensed to UltraSight Inc and may benefit from algorithm commercialization through Mayo Clinic. Dr Pellikka received research support from the American Society of Echocardiography Foundation, Ultromics Ltd, GE Healthcare, and Edwards Lifesciences (all funds paid to Mayo Clinic). Dr Pellikka is also a consultant/adviser for Astellas Pharma. Drs Friedman, Lopez-Jimenez, and Attia are members of the Scientific Advisory Board for Anumana, an artificial intelligence company commercializing AI- electrocardiography. Dr Lopez-Jimenez is a co-inventor of several algorithms using AI- electrocardiography licensed to Anumana and a co-inventor of an artificial heart valve that has been licensed to Colibri Co. The remaining authors have nothing to disclose. Given their roles as Editor-in-Chief and Editorial Board Member, Drs Francisco Lopez-Jimenez, Paul Friedman, and Zachi Attia had no involvement in the peer-review of this article and has no access to information regarding its peer-review. Full responsibility for the editorial process for this article was delegated to an unaffiliated Editor.

## Ethics Statement

All study procedures were approved by the Mayo Clinic institutional review board, and patient cohorts only included individuals who had provided prior authorization for the use of their data in research.
