# Supplementary material for: Leveraging Comprehensive Echo Data to Power Artificial Intelligence Models for Handheld Cardiac Ultrasound
Source: Mayo Clin Proc Digit Health. 2025 Jan 10;3(1):100194. doi: 10.1016/j.mcpdig.2025.100194 (PMC11975991; doi:10.1016/j.mcpdig.2025.100194)
Supplement: Supplementary Data [file mmc1.pdf]

Leveraging Comprehensive Echo Data to Power AI Models for Handheld Cardiac Ultrasound

D M Anisuzzaman, PhD; Jeffrey G. Malins, PhD; John I. Jackson, PhD; Eunjung Lee, PhD; Jwan A. Naser, MBBS; Behrouz Rostami, PhD; Grace Greason, BA; Jared A. Bird, MD; Paul A. Friedman, MD; Jae K. Oh, MD; Patricia A. Pellikka, MD; Jeremy J. Thaden, MD; Francisco Lopez-Jimenez, MD, MSc, MBA; Zachi I. Attia, PhD; Sorin V. Pislaru, MD, PhD; Garvan C. Kane, MD, PhD

Supplementary Appendix

Table of Contents

Supplemental Methods 3

Data acquisition and selection 3

Supplementary Figure 1: The number of studies meeting inclusion and exclusion criteria for the retrospective and prospective cohorts 4

Data processing 5

Model architecture 6

Supplementary Figure 2: Model architecture for the LVEF and age estimation models 7

Model training and hyperparameters 8

Data augmentation 9

Model selection 9

Evaluating model performance 10

Supplemental Results 11

LVEF estimation for the prospective cohort 11

Supplementary Figure 3. Bland-Altman plots showing the difference between model estimates and ground truth labels for point-of-care ultrasound and transthoracic echocardiographic data from the prospective cohort. 11

Age estimation and sex classification models 12

Supplementary Figure 4: Age estimation performance for the AI-ECG and AI-echo age estimation models for the testing dataset from the retrospective cohort of TTE data from Mayo Clinic Rochester. 12

Supplementary Figure 5: Sex classification performance for the AI-ECG and AI-echo sex classification models for the testing dataset from the retrospective cohort of TTE data from Mayo Clinic Rochester. 13

References 14

Supplemental Methods

Data acquisition and selection

For the model development cohort from Mayo Clinic Rochester and surrounding sites in the Mayo Clinic Health System, when randomly sampling from the list of candidate studies, we used the *shuffle* function from version 1.0.2 of the *scikit-learn* library [1] in Python version 3.9.6 [2]. To ensure that the model would be presented with sufficient examples of patients on the middle to lower end of the LVEF distribution, we enriched the sample by supplementing it with batches of patients (randomly drawn from the list of candidate studies) whose LVEF was in the following ranges: less than or equal to 40, between 41 and 45, between 46 and 50, between 51 and 55, or between 56 and 60. From this enriched sample, we excluded TTE studies that included phases which were not coded as baseline/rest (i.e., inspiratory, expiratory, Valsalva).

When splitting studies into the training, validation, and testing datasets, we stratified the sample such that the proportion of data from the lower end of the LVEF distribution (i.e., LVEF ≤ 40) was equivalent across the training, validation, and test datasets. Following this split, as described below, we further applied a view classifier inference score threshold of at least 0.95 for the training and validation datasets but not the testing dataset.

For the testing datasets from Arizona and Florida, studies were randomly drawn from the full set of TTE studies performed at these sites between the dates of January 2022 and February 2024 that met our study constraints (i.e., only a baseline study phase and having an LVEF measurement belonging to the hierarchy of measurements that was used). Importantly, for these cohorts, we did not enrich for the lower end of the LVEF distribution. Similar to the testing cohort from Mayo Clinic Rochester, we did not apply a view classifier inference score threshold. As detailed in Supplementary Figure 1, which illustrates how many studies were

included/excluded at each stage of data selection and preprocessing, we were not able to evaluate some studies because they did not have at least one clip with at least 48 frames for both the A2C and PLAX views.

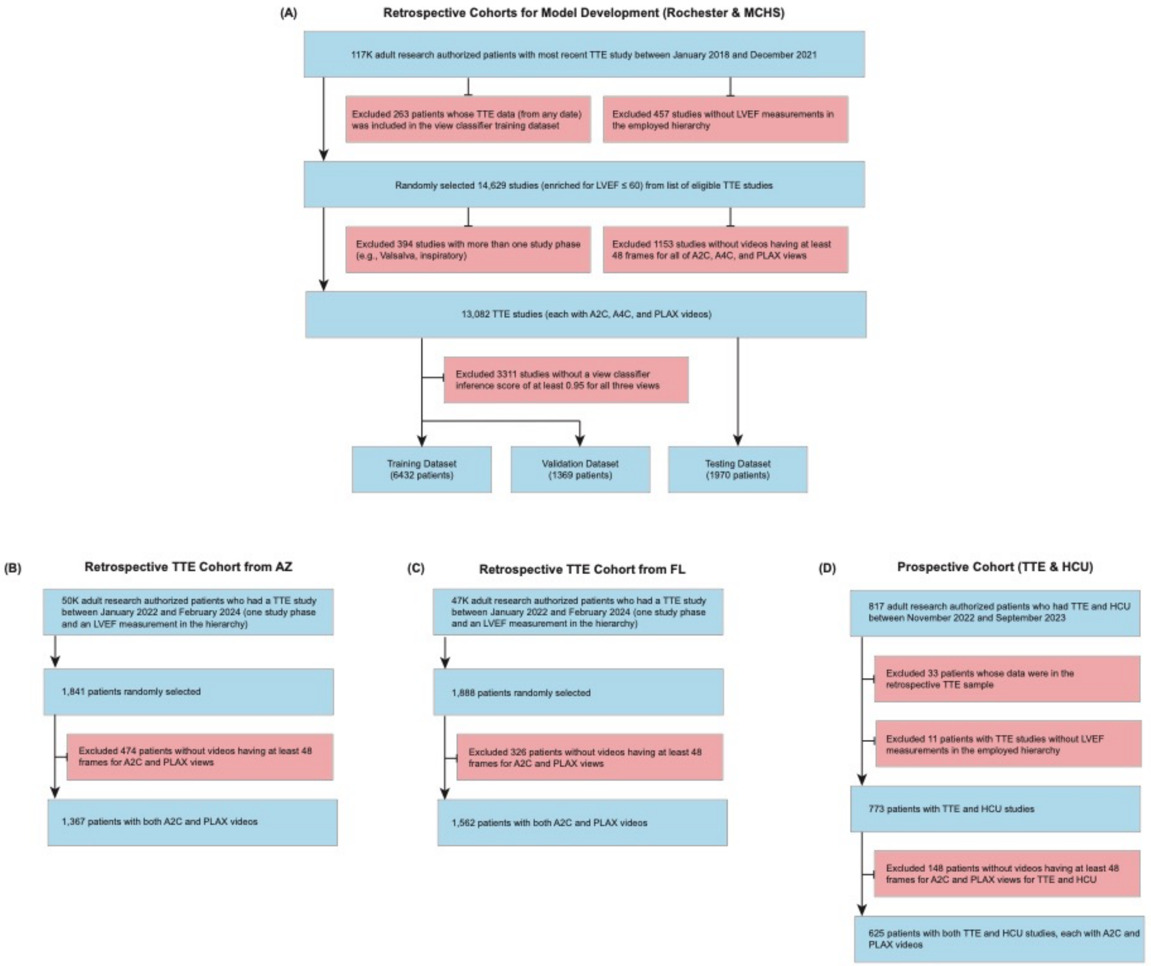

Supplementary Figure 1: The number of studies meeting inclusion and exclusion criteria for (A) the retrospective TTE cohort used to develop and test the LVEF, age, and sex models; (B) the retrospective TTE cohort from AZ used for testing the LVEF model; (C) the retrospective TTE cohort from FL used for testing the LVEF model; (D) the prospective cohort of patients from whom TTE and HCU data were simultaneously collected (used for testing the LVEF, age, and sex models).

Data processing

TTE exams were downloaded using Notion, our internal echocardiographic exam access platform, whereas HCU 2D video clips were downloaded using the web-based platform Q-path (Telexy Healthcare, Port Coquitlam, Canada). Downloaded DICOM images were uncompressed and organized with respect to each patient and exam study date. DICOM videos were loaded using *pydicom* version 2.3.0 [3], and based on the information contained within DICOM headers, candidate videos were selected from each raw data directory that met the following criteria: B-mode clip, at least 48 frames, and not zoomed. For each candidate TTE and HCU video, frames were extracted using an algorithm that isolated the imaging sector (i.e., the 'cone'-shaped portion of a B-mode image) and excluded text information and other labels outside of the imaging sector. This was done by identifying pixels whose intensity values changed over the course of videos. Following this, morphological operations and color-based techniques were used to remove the ECG trace, with context-based filling used to replace missing pixels once the ECG was removed (*opencv* version 4.5.5 in Python [4]). The image was then cropped to fit the imaging sector and padded with columns or rows of zeroes (depending on whether the image width was less than or greater than the height) to generate a square image.

Preprocessed videos were then provided as input to a view classifier deep learning model [5]. This model makes use of a 2-dimensional convolutional neural network based on the ResNet-18 architecture, and provides, for each input frame from a video, a view label as well as the inference score associated with this label. These outputs are then averaged across all frames provided to the model (in this case, ten frames uniformly spaced across either the middle heartbeat for videos with ECG timing information, or across the entire video if ECG timing information was not available). Using the view classifier, the following unenhanced standard

views were extracted: parasternal long axis (abbreviated throughout as PLAX), apical 4-chamber (A4C), and apical 2-chamber (A2C). More specifically, we selected videos from each study that were assigned the highest inference score (averaged across all frames) for each respective view. When selecting these videos, for the training and validation datasets of the retrospective TTE cohort, we used an inference score threshold of 0.95; that is, the inference score for a particular view had to be equal to or greater than 0.95, or else a video was not selected for that view. This threshold was chosen based on some initial experimentation suggesting that this value optimized a tradeoff between data loss and view classification accuracy. Note that a view selection threshold was not applied to either the testing dataset of the retrospective cohort or the TTE and HCU data from the prospective cohort. For selected videos, preprocessed RGB images (corresponding to each frame) were resized (resampled) to 256 x 256 pixels and the three color channels were converted to a single Y (gray-scale) channel, all using OpenCV.

Model architecture

As shown in Supplementary Figure 2, each model had six convolutional blocks and four fully connected blocks. Input data were passed through multiple heads (the number varied depending on the number of views) which fed the model each respective view. The number of convolutional blocks was selected based on a tradeoff between performance and model training time that was observed when we initially evaluated models with shallower (two to five convolutional blocks) and deeper (seven to ten convolutional blocks) architectures. Each convolutional block contains a 3D convolution layer, followed by a batch normalization 3D layer, a ReLU activation, a dropout 3D layer, and a max pooling 3D layer. The kernel and stride size for the temporal dimension were kept smaller than those for the height and width

dimensions for both convolution and pooling, considering that depth for the temporal dimension was smaller than it was for the other two dimensions. The number of channels for the convolutional blocks was 4, 8, 16, 24, 32, and 40, respectively. We also used an increasing amount of dropout on each convolutional layer to mitigate overfitting.

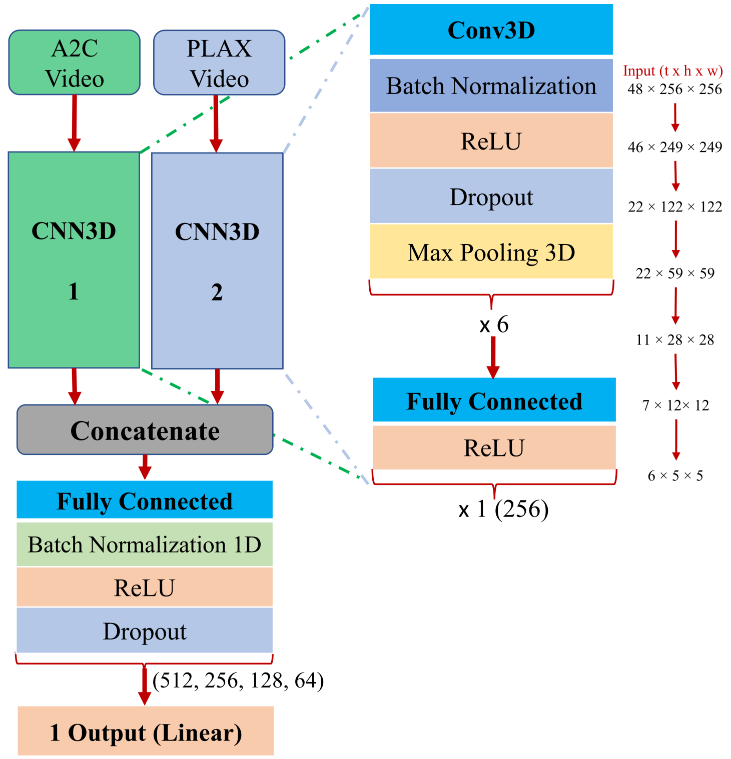

Layers	Convolutions	Channels	Dropout	Max Pooling
1	K: (5,8,8) S: (1,1,1) P: (1,1,1)	4	0.15	K: (1,3,3) S: (1,1,1)
2	K: (5,7,7) S: (2,1,1) P: (1,1,1)	8	0.20	K: (1,3,3) S: (1,2,2)
3	K: (3,6,6) S: (1,1,1) P: (1,1,1)	16	0.25	K: (1,3,3) S: (1,2,2)
4	K: (3,5,5) S: (2,1,1) P: (1,1,1)	24	0.30	K: (1,3,3) S: (1,2,2)
5	K: (3,3,3) S: (1,1,1) P: (0,0,0)	32	0.35	K: (3,3,3) S: (1,2,2)
6	K: (2,2,2) S: (1,1,1) P: (0,0,0)	40	0.40	K: (1,2,2) S: (1,2,2)

K = Kernel (temporal, height, width)
S = Stride (temporal, height, width)
P = Padding (temporal, height, width)

Supplementary Figure 2: Model architecture for the LVEF and age estimation models. The sex classification model was similar in architecture, except the final layer was a SoftMax layer that gave two outputs. Note that initially for LVEF estimation, other model architectures were evaluated including a three-view model (A2C, PLAX, and A4C) that incorporated a third 3D-CNN which processed A4C videos prior to concatenation. However, based on superior performance on the validation dataset, the two-view model taking A2C and PLAX views was selected. These same two views were then used for the age estimation and sex classification models. (A2C, apical 2-chamber; PLAX, parasternal long axis; A4C, apical 4-chamber; CNN, convolutional neural network; Conv3D, 3-dimensional convolutional layer; ReLU, rectified linear unit)

In the case of single-view models, the six convolutional blocks were directly followed by four fully connected blocks prior to the output layer. For multi-view models, each video was

passed through six convolutional blocks, followed by a single fully connected (FC) block with a ReLU activation and 256 units. For multi-view models, blocks were referenced as CNN3D 1 and CNN3D 2 (as well as CNN3D 3, in the case of the three-view model). The outputs of these blocks were concatenated and passed through four FC blocks. Each FC block contained a fully connected layer, followed by a batch normalization 1D layer, a ReLU activation, and a dropout 1D layer. The number of units for each FC layer was 512, 256, 128, and 64, respectively. Finally, for the LVEF and age estimation models, a single linear output layer was used to estimate LVEF or age from a specific video or combination of videos for a specific echocardiographic exam. In contrast, for sex classification, the final output layer was changed to a SoftMax layer that gave a one-hot encoded vector representing a binary output.

Model training and hyperparameters

The rationale for the sliding window technique was that although all the videos in the training dataset (i.e., the retrospective cohort of TTE data) started at the peak of an R-wave, this was not necessarily the case for HCU videos in the prospective dataset. More specifically, due to a lack of ECG timing information, HCU videos started at a random point within the cardiac cycle. Therefore, this method allowed us to address the limitation of requiring a specific starting point for any echocardiographic video, and thereby allowed the model to be more robust to temporal variability in the cardiac cycle. This model feature could also be useful for external datasets lacking temporal synchronicity in terms of the cardiac cycle.

All models were developed using the Python programming language and version 1.12.1 of the PyTorch framework [6]. Models were trained for up to 200 epochs on a fixed timeframe of 6 days and 12 hours due to hardware limitations. Two Tesla V100 GPUs with 32GB memory in

total were used for model training. Trained models were saved based on minimum loss for the validation dataset. We used a batch size of 32, which was divided equally across both GPUs. We started with a learning rate of 0.001 and applied a learning rate scheduler named StepLR [7] that decayed the learning rate of each parameter group by 0.1 every 50 epochs during model training. Optimization was performed using the Adam algorithm [8].

Data augmentation

Central cropping isolated the center of each frame of a video using a 180 x 180 window, with resultant images then resized back to 256 x 256 pixels. Random cropping instead isolated one of the four corners of each frame using the same 180 x 180 window size, with subsequent resizing of the cropped image back to 256 x 256 pixels. Each of the four augmentations were applied to 25% of the training dataset in a random fashion, thus creating an augmented dataset which was introduced to each model alongside the original dataset during model training (i.e., doubling the overall amount of training data). No augmentation was applied to the validation or testing datasets.

Model selection

According to the RMSE score of these seven models for the validation dataset, the ranking was (best to worst): A2C & PLAX, A2C & A4C & PLAX, PLAX, A2C, A4C & PLAX, A2C & A4C, A4C. Therefore, we selected the A2C & PLAX model for all further testing in terms of LVEF.

To estimate patient age and classify patient sex from echocardiogram videos, we developed two separate end-to-end deep learning models. Both the age estimation and sex

classification models followed the same architecture we selected for LVEF estimation (i.e., A2C & PLAX view combined model). However, as mentioned above, whereas the age model architecture was exactly same as it was for the LVEF estimation model, for the sex classification model, we changed the last output layer from a linear output layer with one output to a SoftMax layer that gave a one-hot encoded vector representing a binary output.

Evaluating model performance

To evaluate model performance for LVEF and age estimation (i.e., continuous regression), we computed the root mean square error (RMSE), the Pearson correlation coefficient (r), the coefficient of determination (R^2), and the prediction interval. For LVEF classification and sex classification, we computed the accuracy, sensitivity, specificity, positive predictive value (PPV), negative predictive value (NPV), and area under the receiver operating characteristic curve (AUROC, abbreviated as AUC), with a confidence interval (CI). Detailed explanations regarding these metrics can be found at [9,10].

Supplemental Results

LVEF estimation for the prospective cohort. The Bland-Altman plot in Supplementary Figure 3 illustrates the difference between model estimates and ground truth labels. For both HCU and TTE, model estimates showed a difference of 10% or less from clinically-calculated LVEF in more than 90% of patient exams.

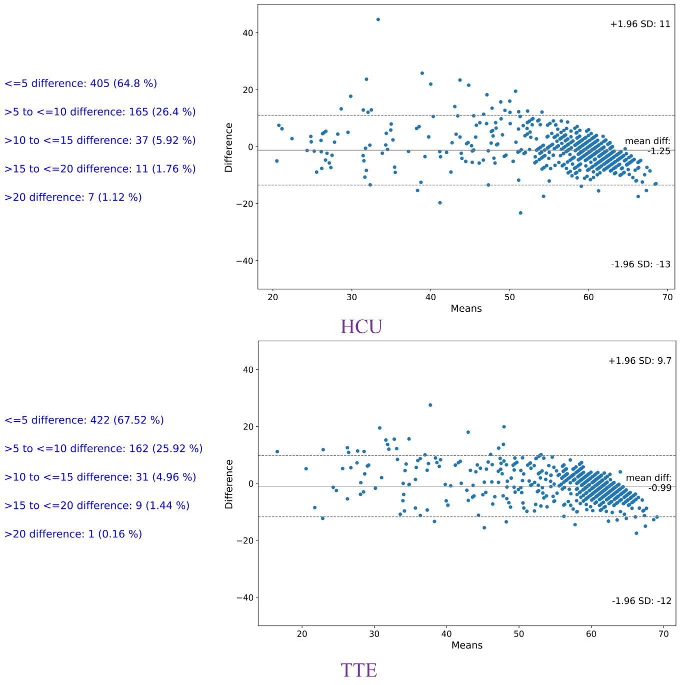

Supplementary Figure 3: Bland-Altman plots showing the difference between model estimates and ground truth labels for point-of-care ultrasound (HCU) (upper panel) and transthoracic echocardiographic (TTE) (lower panel) data from the prospective cohort.

Age estimation and sex classification models

In addition to evaluating the performance of age and sex models using echocardiogram videos, for comparison purposes, we also present results obtained using AI-ECG models previously developed by our team [11]. To do this, we selected a subset of 1346 patients from the test dataset of the retrospective cohort from Mayo Clinic Rochester who had an ECG performed within a 365-day window of their TTE study date, and who were not in the training or validation datasets for the AI-ECG age and sex models. For these 1346 patients, mean age on the TTE study date was 65 (standard deviation = 17; range 18-104), and self-reported sex was female for 42.94% of patients. Note that of these 1346 patients, 1114 had an ECG acquired within a 30-day window.

Supplementary Figure 4 details model performance for the AI-ECG versus AI-echo age estimation models. As shown in the figure, model performance was comparable across all quantified metrics, and a tight correlation was observed when model estimates were directly compared between the AI-echo and AI-ECG models.

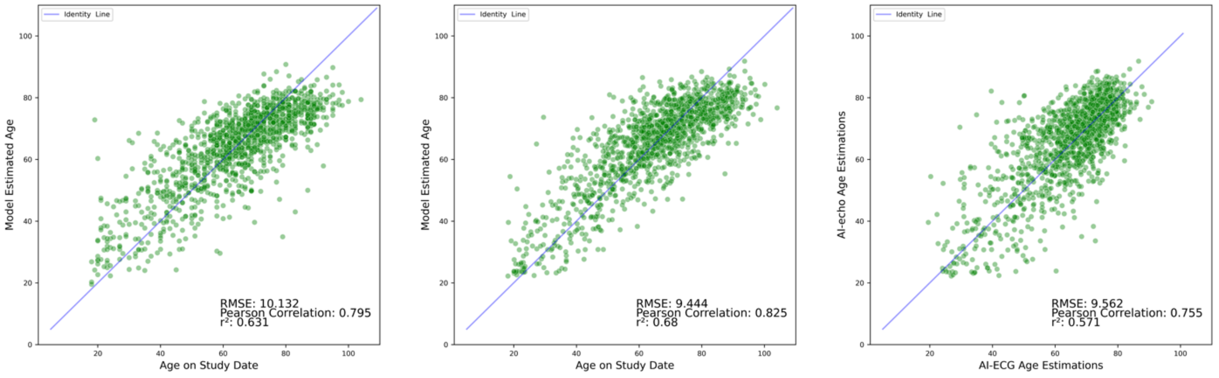

AI-ECG Age Model AI-echo Age Model AI-ECG versus AI-echo Model Estimations (1346)

Supplementary Figure 4: For the test dataset of retrospective TTE data from Mayo Clinic Rochester, age estimation performance for the AI-ECG (left panel) and AI-echo (middle panel) age estimation models, as well as the direct comparison of age estimates between the AI-ECG and AI-echo models (right panel).

Supplementary Figure 5 details model performance for the AI-ECG versus AI-echo sex classification models. As shown in the figure, model performance was comparable across all quantified metrics.

		**Actual**	
		Male	Female
**Predicted**	Male	**589**	69
	Female	179	**509**

Accuracy: 81.6%
Sensitivity: 76.7%
Specificity: 88.1%
PPV: 89.5%
NPV: 74.0%

AI-ECG Sex Model

		**Actual**	
		Male	Female
**Predicted**	Male	**593**	105
	Female	175	**473**

Accuracy: 79.2%
Sensitivity: 77.2%
Specificity: 81.8%
PPV: 85.0%
NPV: 73.0%

AI-echo Sex Model

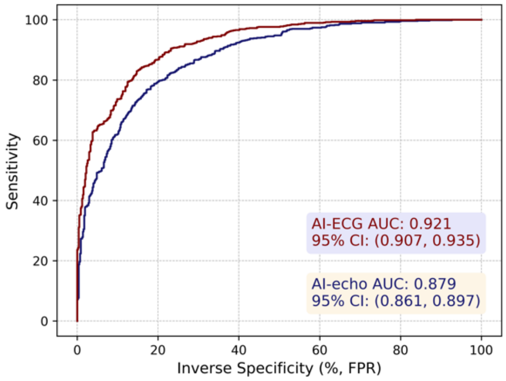

		**AI-echo Model**	
		Male	Female
**AI-ECG Model**	Male	**550**	231
	Female	148	**417**

AI-ECG versus AI-echo Model Estimations (1346)

Supplementary Figure 5: For the test dataset of retrospective TTE data from Mayo Clinic Rochester, sex classification performance for each of the AI-ECG and AI-echo sex classification models as well as a direct comparison between models. For this analysis, male is considered the positive class. (AUC, area under the curve; CI, confidence interval(s); PPV, positive predictive value; NPV, negative predictive value)

References

1. Pedregosa F, Varoquaux G, Gramfort A, Michel V, Thirion B, Grisel O, Blondel M, Prettenhofer P, Weiss R, Dubourg V, et al. Scikit-learn: Machine Learning in Python. *Journal of Machine Learning Research*. 2011;12:2825-2830.
2. Python Software Foundation. Python 3.9.6. https://www.python.org/downloads/release/python-396/. 2021. Accessed May 11.
3. Mason D, scaramallion, mrbean-bremen, rhaxton, Suever J, Vanessasaurus, Orfanos DP, Lemaitre G, Panchal A, Rothberg A, et al. pydicom/pydicom: pydicom 2.3.0 (v2.3.0). Zenodo. 2022. Accessed May 11.
4. Bradski G. The OpenCV Library. *Dr Dobb's Journal of Software Tools*. 2000.
5. Naser JA, Lee E, Pislaru SV, Tsaban G, Malins JG, Jackson JI, Anisuzzaman D, Rostami B, Lopez-Jimenez F, Friedman PA, et al. Artificial intelligence-based classification of echocardiographic views. *European Heart Journal - Digital Health*. 2024;5:260-269. doi: https://doi.org/10.1093/ehjdh/ztae015
6. Paszke A, Gross S, Massa F, Lerer A, Bradbury J, Chanan G, Killeen T, Lin Z, Gimelshein N, Antiga L, et al. Pytorch: An imperative style, high-performance deep learning library. *Advances in Neural Information Processing Systems*. 2019;32.
7. PyTorch Contributors. StepLR. https://pytorch.org/docs/stable/generated/torch.optim.lr_scheduler.StepLR.html. 2023. Accessed May 11.
8. Kingma DP, Ba J. Adam: A method for stochastic optimization. *arXiv preprint arXiv*. 2014;1412.6980.
9. Taylor R. Interpretation of the correlation coefficient: A Basic review. *Journal of Diagnostic Medical Sonography*. 1990;6:35-39.
10. Bewick V, Cheek L, Ball J. Statistics review 13: receiver operating characteristic curves. *Crit Care*. 2004;8:508-512. doi: 10.1186/cc3000
11. Attia ZI, Friedman PA, Noseworthy PA, Lopez-Jimenez F, Ladewig DJ, Satam G, Pellikka PA, Munger TM, Asirvatham SJ, Scott CG, et al. Age and Sex Estimation Using Artificial Intelligence From Standard 12-Lead ECGs. *Circ Arrhythm Electrophysiol*. 2019;12:e007284. doi: 10.1161/CIRCEP.119.007284
